# Transcriptome Profiling Using Single-Molecule Direct RNA Sequencing Approach for In-depth Understanding of Genes in Secondary Metabolism Pathways of *Camellia sinensis*

**DOI:** 10.3389/fpls.2017.01205

**Published:** 2017-07-11

**Authors:** Qingshan Xu, Junyan Zhu, Shiqi Zhao, Yan Hou, Fangdong Li, Yuling Tai, Xiaochun Wan, ChaoLing Wei

**Affiliations:** State Key Laboratory of Tea Plant Biology and Utilization, Anhui Agricultural University Hefei, China

**Keywords:** *Camellia sinensis*, single-molecule sequencing, full-length transcript, alternative splicing, characteristic secondary metabolite

## Abstract

Characteristic secondary metabolites, including flavonoids, theanine and caffeine, are important components of *Camellia sinensis*, and their biosynthesis has attracted widespread interest. Previous studies on the biosynthesis of these major secondary metabolites using next-generation sequencing technologies limited the accurately prediction of full-length (FL) splice isoforms. Herein, we applied single-molecule sequencing to pooled tea plant tissues, to provide a more complete transcriptome of *C. sinensis*. Moreover, we identified 94 FL transcripts and four alternative splicing events for enzyme-coding genes involved in the biosynthesis of flavonoids, theanine and caffeine. According to the comparison between long-read isoforms and assemble transcripts, we improved the quality and accuracy of genes sequenced by short-read next-generation sequencing technology. The resulting FL transcripts, together with the improved assembled transcripts and identified alternative splicing events, enhance our understanding of genes involved in the biosynthesis of characteristic secondary metabolites in *C. sinensis*.

## Introduction

The tea plant (*Camellia sinensis*) is an important horticultural crop and source of one of the most popular natural non-alcoholic beverages consumed across the world (Chen et al., 2007; Zhang et al., 2015). The rich flavors of tea are mainly attributable to the characteristic secondary metabolites including flavonoids, theanine and caffeine (Liang et al., 2001; Mamati et al., 2006). These secondary compounds have been confirmed to be beneficial to human health (Hertog et al., 1993; Cabrera et al., 2006; Khan and Mukhtar, 2007) and contribute to the nutrient content and unique taste of tea (Chu and Juneja, 1997; Chen et al., 2008). Flavonoids such as flavanones, flavones, dihydroflavonols, flavonols, and flavin-3-ols (catechins) are derived from multiple branches of the phenylpropanoid pathway (Dixon and Pasinetti, 2010). Theanine is synthesized from glutamic acid and ethylamine by theanine synthetase (TS) in the roots of the tea plant (Deng et al., 2012). Caffeine is a purine alkaloid that is abundant in the leaves of tea plant (Takeda, 1994; Ashihara et al., 1995). A thorough understanding of the genes underlying the biosynthesis of characteristic metabolites are essential for functional genomic studies.

Due to the large genome size (∼4.0 Gigabases) (Tanaka et al., 2006) of *C. sinensis* and genetic barriers in tea plant tissue culture and transformation, little genomic information is available currently. The genes encoding characteristic secondary metabolite biosynthetic enzymes were mostly discovered through Sanger sequencing (Singh et al., 2008, 2009b) or next-generation sequencing (Shi et al., 2011; Wu et al., 2013, 2014; Wang et al., 2014; Li et al., 2015). Sanger sequencing of full-length (FL) cDNA clones is the most reliable means of transcript discovery, but this method has fallen out of fashion somewhat following the advent of cheaper next-generation sequencing technologies (Wang et al., 2016). Using RNA-seq technology, most of the essential genes that regulate theanine, caffeine, and flavonoid biosynthesis were identified from whole tissues of tea (Shi et al., 2011). By studying transcription profiles of different tissues at different developmental stages, the gene network responsible for the regulation of the secondary metabolic pathways was also elucidated in tea plant (Li et al., 2015). However, the relatively short length of the reads generated from next-generation sequencing prevented to assemble the FL transcripts accurately (Minoche et al., 2014; Dong et al., 2015). Furthermore, in some cases, incorrect annotation can result from the low-quality transcripts generated by short-read RNA-seq sequencing (Au et al., 2012, 2013).

AS is an important post-transcriptional regulatory mechanism in multicellular eukaryotes that significantly enhances transcriptome diversity (Kalsotra and Cooper, 2011; Reddy et al., 2013). Next-generation sequencing revealed that over 60% of multi-exon genes are alternatively spliced in plant, such as *Oryza sativa* (Zhang et al., 2010), *Arabidopsis thaliana* (Marquez et al., 2012), and *Glycine max* (Shen et al., 2014). Up to now, very little was known about the alternative splicing in tea plant for the absence of genome information (Li et al., 2015). Additionally, short reads generated from next-generation sequencing require computational *de novo* assembly, therefore, identification of gene isoforms are not well supported by direct experimental evidence and may suffer from a high incidence of false positives (Au et al., 2012). More recently, single-molecule sequencing (SMS) technology eliminates the need for assembly with much longer reads (Sharon et al., 2013; Tilgner et al., 2014, 2015), providing direct evidence for transcript isoforms of each gene (Au et al., 2013; Chen et al., 2014; Abdelghany et al., 2016). These long-read transcripts can greatly increase the accuracy of transcriptome characterization compared with transcript tags assembled from short RNA-seq reads (Dong et al., 2015). Moreover, the higher error rate associated with SMS sequencing has been addressed by self-correction which involves the use of circular-consensus reads (Li Q. et al., 2014; Xu et al., 2015).

In this study, we employed an SMS approach to generate a more complete/FL transcriptome of *C. sinensis*. Based on long-read databases and genome sequences from bacterial artificial chromosome (BAC) libraries, we acquired FL transcripts and observed alternative splicing events for flavonoid, theanine and caffeine biosynthetic genes. The longer reads improved the quality and accuracy of transcripts generated from short-read assembly. This is the first study to use SMS technology to get the global overview of FL transcripts and alternative splicing events in tea. These results are necessary to deduce the nature of the encoded protein and in assessing a splice variant’s role in gene regulation for *C. sinensis*.

## Materials and Methods

### Plant Materials

Tea plants (*C. sinensis cv. Shuchazao*) were grown in the 916 Tea Plantation in Shucheng County, Anhui Province, China. Eight samples of different tissues were collected from exactly the same tea plant. The tissues sampled were as follows: apical bud, first leaf, mature leaf, old leaf, stems, flowers, fruits and roots. Apical bud, first leaf, mature leaf and stems were collected on June 15, 2015; Old leaf were collected on November 13, 2015; Flowers, fruits and roots were collected on October 12, 2015. All samples were immediately frozen in liquid nitrogen and stored at -80°C until further use.

### RNA Isolation

Total RNA was extracted using the RNeasy Plus Mini kit (Qiagen, Valencia, CA, United States). Total RNA from each sample was quantified and the quality assessed using an Agilent 2100 Bioanalyzer (Agilent Technologies, Palo Alto, CA, United States). Equal amounts of total RNA from each sample were pooled to provide 90 μg of total RNA. PolyA RNAs was isolated from total RNA using Dynal oligo (dT) 25 beads (Invitrogen^TM^ Life Technologies, Carlsbad, CA, United States) according to the manufacturer’s protocol. The isolated polyA RNAs was eluted with 20 μl of RNase-free water and subjected to RNA-seq library construction.

### Library Preparation and Single-Molecule Sequencing

The library was prepared according to the PacBio ISO-Seq experimental workflow (Supplementary Figure S1). The first cDNA strand was synthesized from purified polyA RNAs using a Clontech SMARTer PCR cDNA Synthesis Kit (Clontech, Mountain View, CA, United States). After PCR optimization, large-scale PCR was performed to synthesize second strand cDNA for BluePippin size selection (Sage Science, Inc., Beverly, MA, United States) with size ranges of 0-1 kb, 1-2 kb, 2-3 kb, and 3-6 kb. After size selection, another amplification was performed, and amplified, size selected cDNA products were made into SMRTbell template libraries (0-1 kb, 1-2 kb, 2-3 kb, and 3-6 kb) according to the manufacture’s instruction.

Libraries were prepared by annealing a sequencing primer (SMRTbell Template Prep Kit 1.0) and binding polymerase to the primer-annealed template. Sequencing was performed on a PacBio RS II platform. A total of seven SMRT cells were conducted in this study (Supplementary Table S1).

### Data Analyses of Single-Molecule Sequencing Data

**FIGURE 1:**
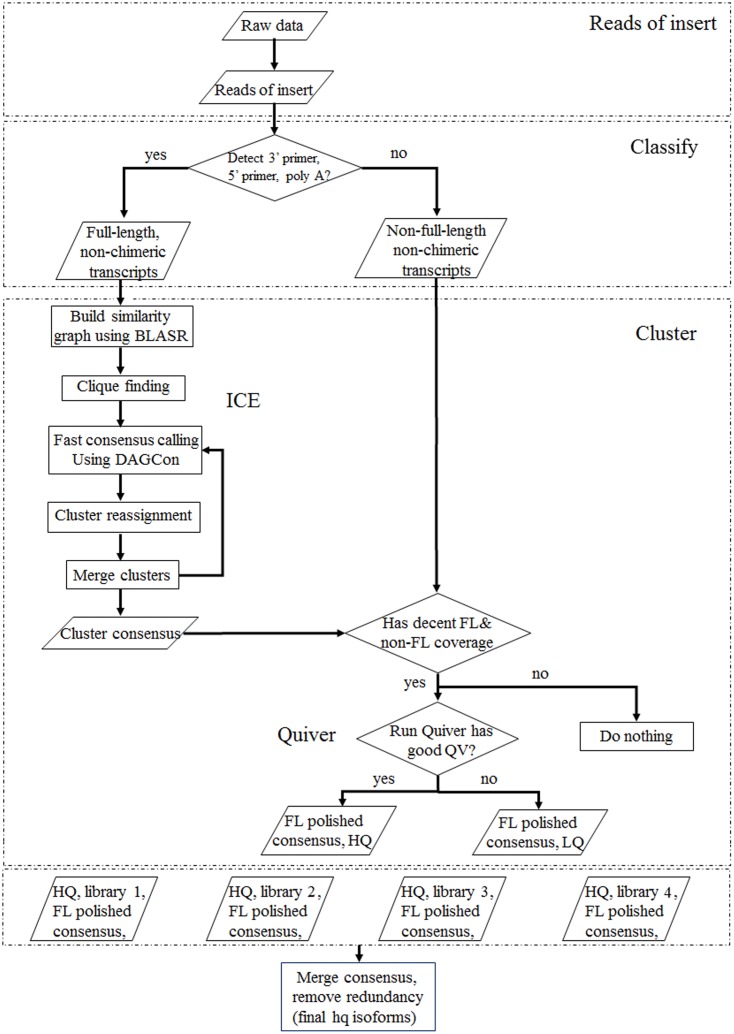
SMRT analysis of each library to obtain high-quality consensus isoforms. Qualified sequencing data produced by Pacific Biosciences RS II were processed using SMRT analysis (Reads of Insert, Classify, Cluster) to obtain consensus full-length isoforms.

Raw data from four libraries produced by Pacific Biosciences RS II were processed following the BGI PacBio transcriptome analysis procedure (SMRT analysis 2.3.0) (**Figure 1**). In this pipeline, the ‘Reads of Insert’ that could either be a FL transcript (as defined by the presence of 5′ primer, 3′ primer, and the polyA tail if applicable) or a non-full-length transcript were generated using a minimum filtering requirement of 0 and a minimum read accuracy of 0.75. In the cluster panel, the options of “Predict Consensus Isoforms using the ICE Algorithm” and “Call Quiver to Polish Consensus Isoforms” were applied to get high quality, FL, and polished consensus transcripts. Finally, the high quality consensus transcripts of multiple libraries were merged together and redundancy removed based on CD-HIT-EST (-c 0.98 -T 6 -G 0 -aL 0.90 -AL 100 -aS 0.98 -AS 30) to obtain final FL isoforms.

### Functional Annotation

Final FL isoforms were searched against NCBI non-redundant (NR), NCBI nucleotide sequence (NT), Swiss-Prot, Cluster of Orthologous Groups (COG) and Kyoto Encyclopedia of Genes and Genomes (KEGG, version 58) databases with a threshold *E*-value ≤10^-5^. Gene Ontology (GO) annotations were determined based on the best BLASTX hit from the NR database using the Blast2GO software version 2.3.5 (*E*-value ≤10^-5^). KEGG pathway analyses were performed using the KEGG Automatic Annotation Server (KAAS^1^).

### Unigene and Isoform Prediction of Major Secondary Metabolites Biosynthetic Genes

To identify candidate genes, isoforms encoding enzymes from characteristic secondary metabolic pathways were clustered using CD-HIT software version 4.6.6 (cd-hit-est *c* = 0.90) (Li et al., 2001). The longest isoform of each cluster was defined as the candidate gene (Li and Godzik, 2006).

Alternative splicing isoforms were analyzed using BLAST^2^ by employing transcripts from each cluster with genome sequences from the BAC library. Alternative splicing isoforms found by BLAST were viewed using the Gene Structure Display Server^3^.

### Validation of Alternative Splicing Isoforms by RT-PCR

For PCR validation of alternative splicing events, 1 μg of total RNA obtained from the eight different tissues was used for reverse transcription (RT) in 20 μl reactions with SuperScript III reverse transcriptase (Invitrogen) and N6 random hexamers (TaKaRa, Dalian, China). Gene-specific primers were designed with Primer Premier 6 to span the predicted splicing events (Supplementary Table S2). PCR was performed as follows: 3 min at 94°C, followed by 35 cycles of 94°C for 30 s, 55°C for 30 s, and 72°C for a time period proportional to the predicted product size. PCR amplification was monitored by 2.5% agarose gel electrophoresis.

PCR products were excised from the gel and purified using a gel extraction kit (Qiagen, Hilden, Germany). Purified products were cloned into the pGEM-T easy vector (Promega, United States) and plasmids were isolated using the Qiagen plasmid mini-isolation kit and confirmed by sequencing. Sequences were aligned with related isoforms to confirm the predicted alternative splicing isoforms.

### Comparison with Short-Read Assemblies

Short-read sequences based on Illumina Hiseq2000 sequencing were selected for comparison with *C. sinensis* FL transcripts. Illumina data were obtained from same eight tea plant (*C. sinensis cv*. *Shuchazao*) tissues (buds, first leaf, mature leaf, old leaf, stems, flowers, fruits and roots) in our previous study (unpublished data). Clean reads for each tissue were assembled and annotated to generate unigenes, which were merged into the final dataset and redundancy removed by CD-HIT-EST (-c 0.98 -T 6 -G 0 -aL 0.90 -AL 100 -aS 0.98 -AS 30).

Candidate secondary metabolic pathway genes were identified using CD-HIT software (cd-hit-est *c* = 0.90) (Li and Godzik, 2006). Comparison of FL and Illumina-derived candidate secondary metabolic pathway genes was performed using local BLASTN (1e^-10^ cut-off).

## Results

### High Quality Reads Were Obtained from *Camellia sinensis* by Full-Length Sequencing

To identify as many isoforms as possible, eight different *C. sinensis* tissues were harvested for RNA isolation. Equal amounts of total RNA from each tissue were pooled together and reverse-transcribed. To minimize bias that favors sequencing of shorter transcripts, multiple size-fractionated libraries (<1, 1–2, 2–3 and 3–6 kb) were made using BluePippin. Four ISO-Seq libraries were constructed for one sample, and seven cells were sequenced using the Pacific Bioscience RS II platform, generating 361,947 reads. The mean read lengths of inserts from different libraries (<1, 1–2, 2–3, and 3–6 kb) produced by SMS sequencing were 768, 2160, 3023, and 3885 bases, respectively (Supplementary Table S1).

**FIGURE 2:**
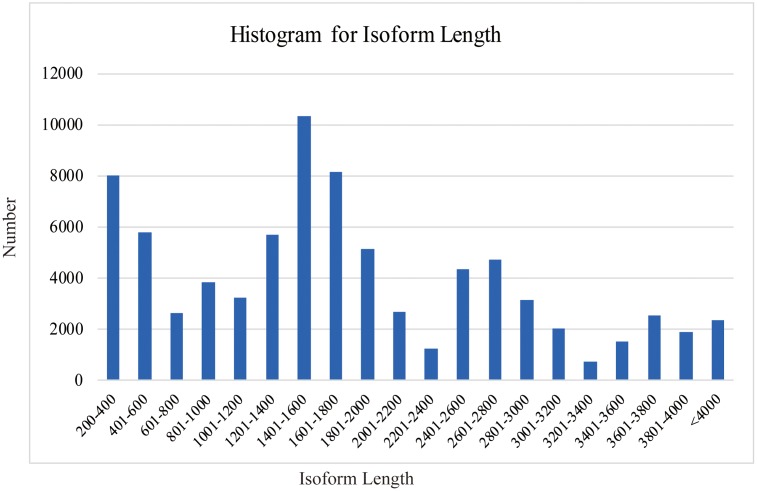
Length distribution of *C. sinensis* transcripts.

SMRT analyses (Reads of Insert, Classify and Cluster) were used to obtain high-quality consensus isoforms (**Figure 1**). Reads of Insert from different libraries (<1, 1–2, 2–3, and 3–6 kb) were classified into 38,131, 83,638, 64,244 and 24,669 FL non-chimeric transcripts, respectively, depending on whether 5′ and 3′ primer sequences or polyA tails were detected (Supplementary Table S3). ICE and Quiver were then used to cluster and polish the non-chimeric transcripts. After clustering and polishing, 21,093, 34,891, 26,633, and 9,021 high quality, FL, and polished consensus transcripts were generated for the four libraries, respectively (Supplementary Table S4). The quality distribution of consensus isoforms were closed to 1 (Supplementary Figure S2). Finally, 91,638 high-quality consensus isoforms of the four libraries were merged into 80,217 isoforms with an average length of 1,781 bp and N50 of 2,459 bp (**Table 1**). In total, 68,360 isoforms (85.2%) were longer than 500bp, and 59900 isoforms (74.7%) were longer than 1 kb (**Figure 2**).

**Table 1:** Summary of final *C. sinensis* consensus isoforms.

Sample	Total isoforms	Total bases (bp)	Mean length (bp)	N50 (bp)
Total	80,217	142,878,553	1,781	2,459

### Functional Annotation and Categorization of the Isoforms

**FIGURE 3:**
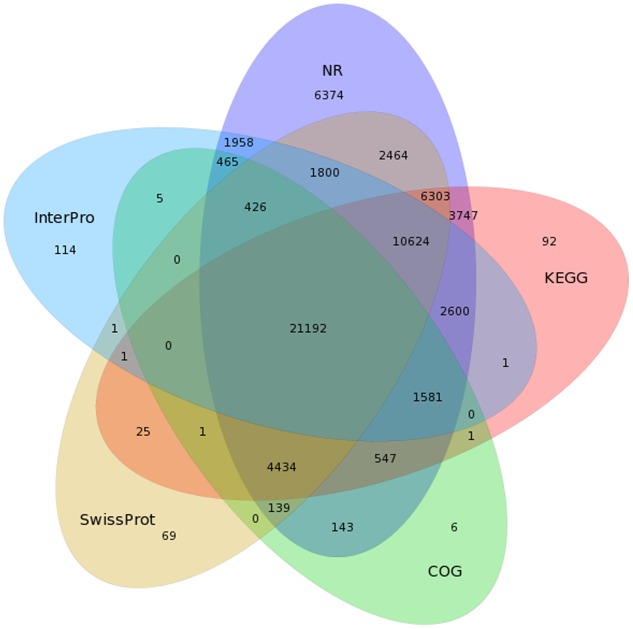
Venn diagram of NR, COG, KEGG, SwissProt, and InterPro results for the *C. sinensis* transcriptome.

To predict and analyze the function of the 80,217 isoforms, we use BLAST (Altschul et al., 1990), BLAST2GO (Conesa et al., 2005), and InterProScan 5 (Quevillon et al., 2005) to perform functional annotation (using NR, NT, SwissProt, KEGG, COG, GO, and InterPro databases). A total of 72,877 isoforms were successfully matched to known proteins in at least one out the five databases, and 21,192 isoforms received high scores with proteins in all five databases (**Figure 3** and **Table 2**).

**Table 2:** Summary of functional annotation results for *C. sinensis* transcripts.

		Nr	Nt	SwissProt	KEGG	COG	InterPro	GO	
Values	Total	annotated	annotated	annotated	annotated	annotated	annotated	annotated	Overall
Number	80,217	64,797	69,456	47,479	51,149	28,940	40,768	15,119	72,887
Percentage	100%	80.78%	86.59%	59.19%	63.76%	36.08%	50.82%	18.85%	90.86%

To functionally classify the *C. sinensis* transcripts, GO terms were assigned to each isoform using BLAST2GO based on the best BLASTx hit from the NR database. In total, 15,119 isoforms were assigned GO terms, which were classified into three major categories (molecular function, cellular component and biological process; Supplementary Figure S3). For molecular function classification, major categories were “catalytic activity” (GO: 0003824) and “binding” (GO: 0005488). In the cellular component category, isoforms involved in the “cell part” (5,977, 39.5% of the total), “cell” (5,977, 39.5%) and “organelle” (4,278, 28.3%) were highly represented. The major subgroups of biological processes were “cellular process” (GO: 0009987) and “metabolic process” (GO: 0008152).

Cluster of Orthologous Group contains protein sequences encoded in 21 prokaryotic and eukaryotic genomes, and this database was used to evaluate the completeness of the isoforms and the validity of the annotations. A total of 28,940 isoforms were assigned to 25 functional clusters (Supplementary Figure S4). “General function prediction only” (24.8%, 7,177), “replication, recombination and repair” (15.5%, 4,484), “transcription” (14.4%, 4,165), “post-translational modification, protein turnover, chaperones” (12.2%, 3,528), and “signal transduction mechanisms” (11.0%, 3,169) were the five largest categories. Secondary metabolites are very important for the taste and quality of tea, and approximately 4.1% (1,178) of isoforms were clustered into the secondary metabolism category (Supplementary Figure S4).

In order to explore the biological functions and interactions of genes in *C. sinensis*, isoforms were searched against the KEGG database. A total of 51,149 isoforms were annotated and assigned to 135 functional categories (**Table 2** and Supplementary Figure S5). Among these pathways, the “biosynthesis of secondary metabolites” pathway included 2176 isoforms, providing a valuable resource for further gene function research.

### Unigenes and Isoforms in Flavonoid Pathway

**FIGURE 4:**
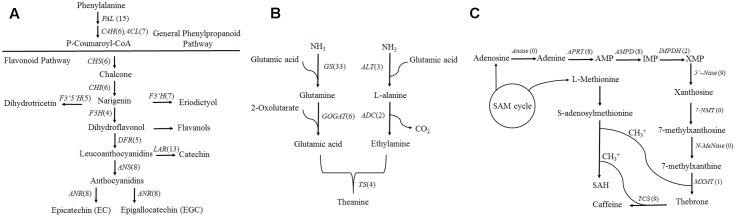
*Camellia sinensis* transcripts involved in three secondary metabolite biosynthetic pathways. Numbers in brackets following each gene indicate the number of unigenes identified in C. *sinensis*. **(A)** Flavonoid biosynthesis pathway. **(B)** Theanine biosynthesis pathway. **(C)** Caffeine biosynthesis pathway.

Based on the KEGG database, a total of 301 isoforms were observed in flavonoid pathway (Supplementary Data S1) clustering into 90 candidate genes by CD-HIT-EST (*c* = 0.90) software. Flavonoids are synthesized via the phenylpropanoid pathway by the enzymes phenylalanine ammonia lyase (*PAL*), cinnamate 4-hydroxylase (*C4H*) and 4-coumarate CoA ligase (*4CL*). Fifteen, six and seven genes were annotated as *PAL*, *C4H* and *4CL*, respectively (**Figure 4A**). Among them, fifteen *PAL* were generated from 37 isoforms, and the isoforms “tea17336” and “tea3529” in different clusters may be transcribed from the same gene by alternative splicing (Supplementary Figure S6). Furthermore, *PAL* isoforms, “tea20264,” “tea22666” and “tea19184” shared significant similarity with *CSPAL* (GenBank accession number: AY694188) which was associated with catechin accumulation (Singh et al., 2009a), and *PAL* isoform tea22927 showed 81.7% identity with *PtPAL2* (GenBank accession number: AF480620) which was expressed in heavily lignified structural cells of Quaking Aspen shoots (Kao et al., 2002).

**FIGURE 5:**
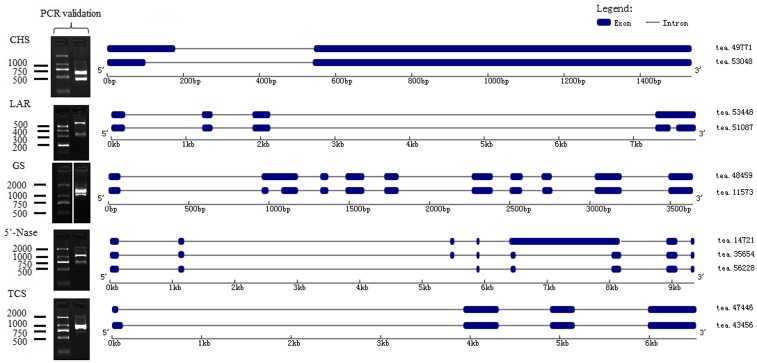
Alternative splicing (AS) isoforms of characterized genes covered by PacBio long reads.

Chalcone synthase (*CHS*) is the first enzyme of the general flavonoid pathway, and this enzyme mediates the influx of substrate from the phenylpropanoid pathway. By mapping isoforms to genome sequences in the BAC library, *CHS* (tea49771 and tea53048) were characterized as alternative 5′ splice sites (**Figure 5**). Subsequently, the stereo-specific cyclization of chalcones into naringenin is catalyzed by chalcone isomerase (*CHI*). Flavonoid 3′-hydroxylase (*F3*′*H*) and flavonoid 3′, 5′-hydroxylase (*F3*′*5*′*H*) catalyze the formation of eriodictyol and dihydrotricetin from naringenin (Wang et al., 2014). Five *F3*′*5*′*H* (tea27534, tea24671, tea22363, tea35097, and tea43773) genes were obtained from 18 isoforms in the present study. Of them, one gene (tea43773) with isoform (tea27827) from another gene (tea24671) cluster may undergo alternative splicing events (Supplementary Figure S6). A BLAST search of “tea27827” and “tea24671” revealed 98.5 and 98.4% identity with *CSF3*′*5*′*H’* (GenBank accession number: DQ194358) which played a critical role in the accumulation of tea catechins (Wang et al., 2014).

The formation of flavan-3-ols (e.g., catechin and gallocatechin) can be produced from leucoanthocyanidins by leucoanthocyanidin reductase (*LAR*) (Liu et al., 2016). Of the 13 *LAR* genes identified in this study, “tea53448” and “tea51087” were characterized as intron retention sites. Moreover, some *LAR* isoforms (tea51293, tea55264, tea51087, and tea51953) in our database shared significant similarity with *CSLAR* gene (GenBank accession no. GU992401) whose overexpression in tobacco leading to the accumulation of higher levels of epicatechin and its glucoside than of catechin (Pang et al., 2013). The generation of epi-flavan-3-ols (epicatechin and epigallocatechin) were achieved through a two-step reaction of leucoanthocyanidin catalyzed by leucoanthocyanidin oxidase (*ANS*) and anthocyanidin reductase (*ANR*) (Li et al., 2015). There were eight *ANS* genes and eight *ANR* genes from the long-read transcripts. Among them, tea52647 and tea52640 from different clusters were fell into the intron retention class (Supplementary Figure S6). Furthermore, by aligning long-read sequences with complete CDS data from NCBI, 50 genes were designated as FL transcripts (Supplementary Data S2).

### Unigenes and Isoforms in Theanine Pathway

In total, 123 isoforms involved in theanine biosynthesis were annotated by the KEGG database (Supplementary Data S1). Based on clustering analysis with CD-HIT-EST (*c* = 0.90), 44 candidate genes were identified from these isoforms and 28 genes were considered to be FL transcripts followed by the alignment of long-read sequences with complete CDS data from NCBI (Supplementary Data S2).

Theanine is synthesized from glutamic acid and ethylamine via *TS*, alanine transaminase (*ALT*), arginine decarboxylase (*ADC*), glutamine synthetase (*GS*), glutamate synthase (*Fe-GOGAT*), and glutamate dehydrogenase (*GDH*) (Li et al., 2015). There were thirty-three *GSs/TSs*, four *GOGATs (NADPH)*, two *GOGATs (Fe)*, three *ALTs* and two *ADCs* in our database (**Figure 4B**). Of 33 *GSs/TS* genes, tea48459 and tea 11573 were characterized as intron retention by mapping isoforms to genome sequences of the BAC library (**Figure 5**). Additionally, sequence analysis of *GS* isoforms tea47901, tea53333 and tea43896 revealed 99.5, 99.1, and 99.0% identity with *CsGS* (Genbank accession No. EF055882) whose expression was stimulated in response to abscisic acid, salicylic acid, and hydrogen peroxide in tea plant (Rana et al., 2008).

### Unigenes and Isoforms in Caffeine Pathway

In our database, 105 isoforms annotated by KEGG database in caffeine pathway were clustered into 37 candidate genes by CD-HIT-EST (*c* = 0.90) (Supplementary Data S1). The caffeine biosynthesis pathway is part of purine metabolism and comprises purine biosynthesis and purine modification steps (Zrenner et al., 2005). Purine biosynthesis starts from adenosine, and involves adenosine nucleosidase (*Anase*), adenine phosphoribosyltransferase (*APRT*), AMP deaminase (*AMPDA*), IMP dehydrogenase (*IMPDH*), and 5′-nucleotidase (*5′-Nase*). Eight *APRTs*, eight *AMPDs*, two *IMPDHs* and nine *5′-Nases* were identified in the present study. Among them, nine *5′-Nase* were yielded from 15 isoforms. Of the 15 isoforms tea14721, tea35654 and tea56228 from the same cluster appeared to undergo exon skipping and intron retention (**Figure 5**). Moreover, tea28112 and tea31199 from different *5′-Nase* were characterized as exon skipping (Supplementary Figure S6).

Purine modification steps include one nucleosidase reaction and three methylations (Shi et al., 2011). Caffeine is derived from xanthosine (*XR*) via 7-methylxanthosine synthase (*7-NMT*), N-methylnucleotidase (*N-MeNase*), theobromine synthase (*MXMT*), and tea caffeine synthase (*TCS*). There were one *MXMT* and nine *TCSs* in our database (**Figure 4C**). Among nine *TCSs*, tea47446 and tea43456 were identified as alternative 5′ splicing event (**Figure 5**). Another *TCS* isoforms (tea39349 and tea26065) from different clusters appeared to undergo intron retention (Supplementary Figure S6). In addition, 16 genes were detected as FL transcripts by aligning long-read sequences with complete CDS data from NCBI (Supplementary Data S2).

### PacBio Isoforms Improved the Quality of Transcripts from Short-Read Assembly

A total of 208 short-read transcripts annotated by KEGG database were obtained from our previous study (Supplementary Data S1), of which 143, 35, and 30 transcripts were involved in flavonoid, theanine and caffeine biosynthesis, respectively. These 208 transcripts were then clustered into 147 candidate genes by CD-HIT-EST (*c* = 0.90), including 105 genes in the flavonoid pathway, 18 genes in the theanine pathway, and 24 genes in the caffeine pathway (Supplementary Data S1).

**FIGURE 6:**
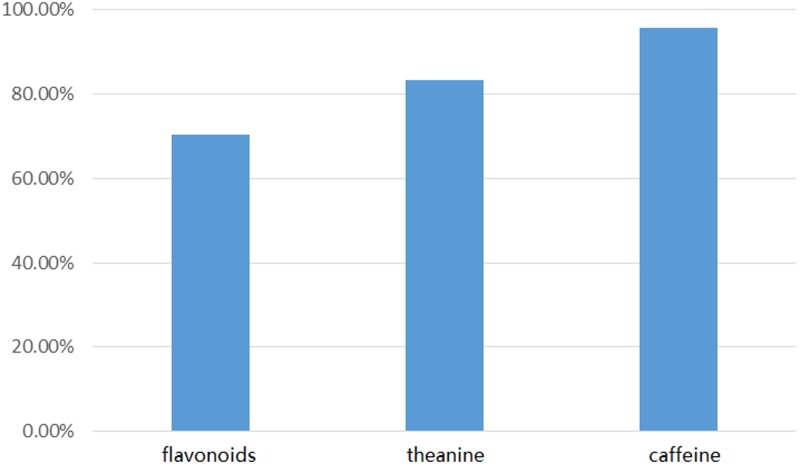
BLAST comparison of Short-Seq sequences and long-read data.

We compared 147 candidate genes from Illumina sequencing with our 171 long-read genes using local BLASTN. The comparison revealed a good agreement between the short-read unigenes and the long-read database at the nucleotide level (**Figure 6** and Supplementary Data S3). For the flavonoid pathway, we identified 74 (70.5%) unigenes from Short-Seq with a high degree of consistency with our database. For the theanine pathway, 15 (83.3%) short-seq unigenes were mapped to 10 long-read genes with high homology. For the caffeine pathway, 23 (95.8%) short-seq unigenes shared significant homology with 17 long-read genes.

**FIGURE 7:**
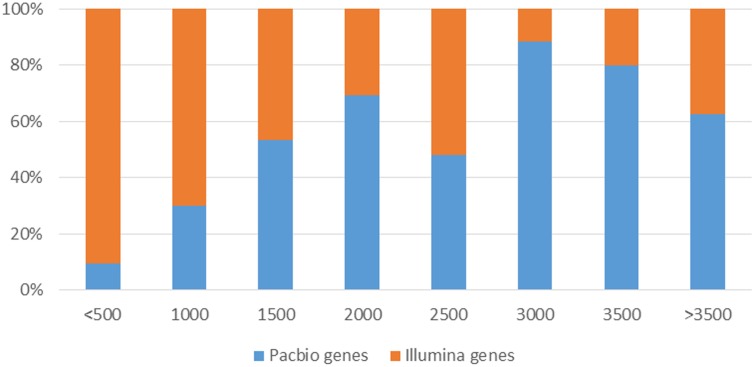
Comparison of transcript length distribution from different sequencing platforms.

However, SMS-Seq genes were longer than Short-Seq unigenes. Approximately 25.9% of the assembled unigenes from short-seq reads were <500 bases, whereas only 2.3% of the isoforms from the PACBIO reads were <500 bases (**Figure 7**). Notably, many of these short-seq unigenes were completely mapped to the same genes of long-read sequences. For example, CL9445.Contig2 (ANS), CL9445.Contig3 (ANS), Unigene6902 (ANS), and Unigene22214 (ANS) were all mapped to tea48033 (ANS) with 100% homology (Supplementary Figure S7). These results suggest that most candidate genes assembled from the Short-Seq reads did not represent FL cDNAs. Our long-read data therefore improved the quality of transcripts assembled from Illumina short reads.

In addition, a few SMS genes shared significant homology with much longer Short-Seq unigenes. Interestingly, these Short-Seq unigenes included two complete CDS regions encoding two identical or different genes, whereas the related SMS genes were only homologous to part of the Short-Seq unigenes. For instance, tea5819 sequences were completely mapped to CL10428.Contig2, and the overlapping regions share the same complete CDS as GOGAT-Fe. However, the region of CL10428.Contig2 without SMS sequence coverage includes another complete CDS region encoding the ATP-dependent RNA helicase-like protein DB10. Tea9026 (GOGAT-Fe) sequences were mapped to two regions of CL10260.Contig7 that have the same CDS region encoding GOGAT-Fe (data not shown). This result suggests that transcripts generated from Short-Seq data may be susceptible to misassembly.

### Validation of Alternative Splicing Events Identified by Multiple Alignment

To experimentally confirm the accuracy of the identified alternative splicing isoforms, three genes involved in flavonoid, theanine and caffeine biosynthesis annotated as a single transcript but present as two or more isoforms were selected for RT-PCR analysis. Primers were designed and synthesized (Supplementary Table S2) and used for RT-PCR using RNA from seven different tissues. The results showed that size of the fragments and the bands on the agarose gel were consistent with the alternative splicing isoforms (**Figure 5**). We cloned the DNA fragments corresponding to the predicted sizes and verified the isoforms by sequencing. Sequences and alignment of the verified isoforms are shown in Supplementary Data S4.

## Discussion

High-throughput mRNA sequencing studies using next-generation sequencing technologies have opened up a new era of transcriptome-wide research (Au et al., 2012; Mutz et al., 2013). Such approaches are particularly suitable for transcription profiling in non-model organisms that lack genomic sequences (Kawaharamiki et al., 2011; Shi et al., 2011). To date, most *C. sinensis* transcript studies have been based on next-generation sequencing (Wu et al., 2014; Zhang et al., 2015), and the short reads resulting from this approach have prevented the accurate assembly of FL transcripts in the absence of genomic sequence information (Au et al., 2013). In the present study, several Short-Seq reads from next-generation sequencing can be completely aligned to the same gene in our dataset (Supplementary Figure S7). Moreover, some misassembly transcripts were found by comparing with the long-read isoforms in our dataset. This result confirmed previous studies that transcripts generated from next-generation sequencing may suffer from misassembly (Schliesky et al., 2012; Li B. et al., 2014) and long reads produced by SMS sequencing technology can facilitate gene identification and annotation (Dong et al., 2015).

Previous studies have demonstrated the ability of SMS sequencing technology to generate continuous long reads (Abdelghany et al., 2016; Wang et al., 2016; Xu et al., 2016). Similar results were also observed in our study, resulting 80,217 isoforms with an average length of 1,781 bp were directly obtained by using SMS (Supplementary Table S5). By contrast, 55,088 transcripts were assembled and annotated from mixed tissue samples of *C. sinensis* based on next-generation sequencing, with an average unigene length of 355 bp (Shi et al., 2011). A total of 347,827 assembly transcripts were yielded from 13 different tea samples of various organs and developmental stages, with an average size of 791.2 bp (Li et al., 2015). On the other hand, we also identified 94 FL transcripts involved in the biosynthesis of flavonoids, theanine and caffeine by employing NCBI complete CDS. The above evidence indicated our results included a large number of longer transcripts specific to *C*. *sinensis* with known functions, which will be useful for improving the accuracy and quality of *C.* *sinensis* transcripts.

Due to its advantages, such as the highly accurate reads and the low costs, Illumina based RNA-seq is widely used for transcriptome analysis (Liu et al., 2012). However, for the alternative splicing events analysis, short reads require additional computational *de novo* assembly, therefore, it is difficult to infer the accuracy of gene model prediction (Steijger et al., 2013; Tilgner et al., 2013; Wang et al., 2016). These limitations were overcome with the emergence of SMS sequencing technology which can generate kilobase-sized sequencing reads in the absence of PCR amplification, where one read usually represents one FL transcript (Sharon et al., 2013; Gordon et al., 2015). In this research, alternative splicing events of some characteristic metabolic genes was predicted followed by the alignment with the BAC library, and confirmed by RT-PCR and sanger sequencing (**Figure 5**). Our results demonstrated that SMS sequencing is highly powerful in alternative splicing event discovery and provides a rich data resource for later functional studies of different isoforms in *C. sinensis*.

## Conclusion

In summary, we identified numerous long-read isoforms specific to *C. sinensis* and characterized FL transcripts and alternative splicing events related to flavonoid, theanine and caffeine biosynthesis. The availability of FL isoforms can improve *C. sinensis* transcriptome characterization. The identification of alternative splicing events can deduce the nature of the encoded protein and in assessing a splice variant’s role in gene regulation for *C. sinensis*. Furthermore, the FL transcripts generated in our study provide a more accurate depiction of gene transcription and will greatly improve *C. sinensis* genome annotation in the future.

## Accession Codes

Raw data and 529 isoforms generated from SMRT sequencing and 208 transcripts produced by ILLUMINA HiSeq have been submitted to the Sequence Read Archive (SRA) of the National Center for Biotechnology Information (NCBI) under accession numbers SRR5460108 and SRX2433645.

## Author Contributions

CW and XW conceived and designed the study. QX analyzed the data and wrote the manuscript. JZ performed PCR validation experiments. YH, FL, and SZ given the advice for data analyzing. YT provided bacterial artificial chromosome libraries. All authors have read and approved the final version of the manuscript.

## Conflict of Interest Statement

The authors declare that the research was conducted in the absence of any commercial or financial relationships that could be construed as a potential conflict of interest.

## Footnotes

1 www.genome.jp/tools/kaas/

2 https://blast.ncbi.nlm.nih.gov/Blast.cgi

3 http://gsds.cbi.pku.edu.cn/
