# Supplementary material for: Transcriptome Profiling Using Single-Molecule Direct RNA Sequencing Approach for In-depth Understanding of Genes in Secondary Metabolism Pathways of Camellia sinensis
Source: Front Plant Sci. 2017 Jul 11;8:1205. doi: 10.3389/fpls.2017.01205 (PMC5504172; doi:10.3389/fpls.2017.01205)
Supplement: Supplementary file 1 [file Presentation_1.PDF]

Supplementary information

Supplementary Table 1 Summary of project sequencing overview of *C. sinensis*

Sample	Library	Cell Number	Reads of Insert	Read Bases of Insert (bp)	Mean Read Length of Insert (bp)	Mean Read Quality of Insert	Mean Number of Passes
tea	<1k	2	55,037	42,298,256	768	0.96	33
tea	1–2k	2	135,732	293,235,156	2,160	0.94	11
tea	2–3k	2	119,629	361,701,882	3,023	0.91	9
tea	3–6k	1	51,549	200,280,602	3,885	0.88	4

Supplementary Table 2. Sequences of primers used for validation of splicing

**Gene Name (ID)**	**Left primer(5'-3')**	**Right primer(5'-3')**
CHS (tea.49771)	AAATGGTCACTGTCGAGG	CTCCATCACCAAATAGGG
CHS (tea.53048)	CAGAGCGACAAATCAATG	CTCCATCACCAAATAGGG
GS (tea.11573)	AACCTTTCGGACACTACG	CCCAACCAAACATAGCATAA
GS (tea.48459)	AGAAGCAAAGCAAGGGTAATA	ACAATGAAACAGACAGAGC
TCS (tea.47446)	AGACAGAGATGAAGGAGG	TGG CAA CCG TGG CAA AC
TCS (tea.45436)	CTTCTTTCACAGTAAGATAATG	AAGATAGCCTACCACGAAG
5’-Nase (tea.14721)	GATTGGTATGTAGACTCTTTC	ATGATGGGTAAGCAAGCC
5’-Nase (tea.35654)	TTTGCAGTTTCTGGAACC	AAAGAACCACCTGAAAGC
5’-Nase (tea.56228)	TTTCTGGTAATCAGCGGC	GAAGTAAGCCTGGCAATC
LAR (51087)	AAGAAGATTGGACGCACTC	TTTCCATCCGTCTCATCTG
LAR (53448)	CAGCAGCAGCAGGTTAAAT	TCTTCCCACTGAAACAATAC
AMPD (10087)	AAAGACGCCCTACGAAAC	TGGTGAGAAGAAATAGTGAG
AMPD (9259)	ATGGATACCTATGCTGTTC	ATATTGGGATGGATCTAGC

Supplementary Table 3 Reads of insert classify summary of *C. sinensis*

Sample	Library	reads of insert	five prime reads	three prime reads	poly-A reads	full-length non-chimeric reads	full-length non-chimeric read length(bp)
tea	<1k	55,037	44,515(80.9%)	46,648(84.8%)	45,758(83.1%)	38,131(69.28%)	604
tea	1–2k	135,732	94,081(69.3%)	99,754(73.5%)	99,015(73.0%)	83,638(61.62%)	1635
tea	2–3k	119,629	74,782(62.5%)	79,771(66.7%)	79,152(66.2%)	64,244(53.7%)	2409
tea	3–6k	51,549	28,824(55.9%)	32,092(62.3%)	31,661(61.4%)	24,669(47.86%)	3861

Supplementary Table 4 Cluster summary for each library of *C. sinensis*

Sample	Library	Cluster type	Total isoforms	Total base (bp)	Mean Quality	Mean isoform length (bp)
tea	1–2k	High quality	34,891	56,455,912	0.9965	1,618
tea	1–2k	Low quality	10,268	18,557,051	0.6815	1,807
tea	2–3k	High quality	26,633	59,014,478	0.9954	2,216
tea	2–3k	Low quality	16,330	43,708,641	0.7463	2,677
tea	3–6k	High quality	9,021	34,571,978	0.9906	3,832
tea	3–6k	Low quality	9,969	39,107,814	0.6296	3,923
tea	<1k	High quality	21,093	12,370,182	0.9954	586
tea	<1k	Low quality	3,618	28,161,76	0.6387	778

Supplementary Table 5 Comparison of PacBio isoforms with short-read assembly

Sample	Total transcripts	Annotatd transcripts	Mean Length (bp)	N50 (bp)
Pacbio (This study)	80,217	72,877	1,781	2,459
short-read (Shi 2011)	127,094	55,088	355	506
short-read (Li 2014b)	347,827	176,356	791.2	1340

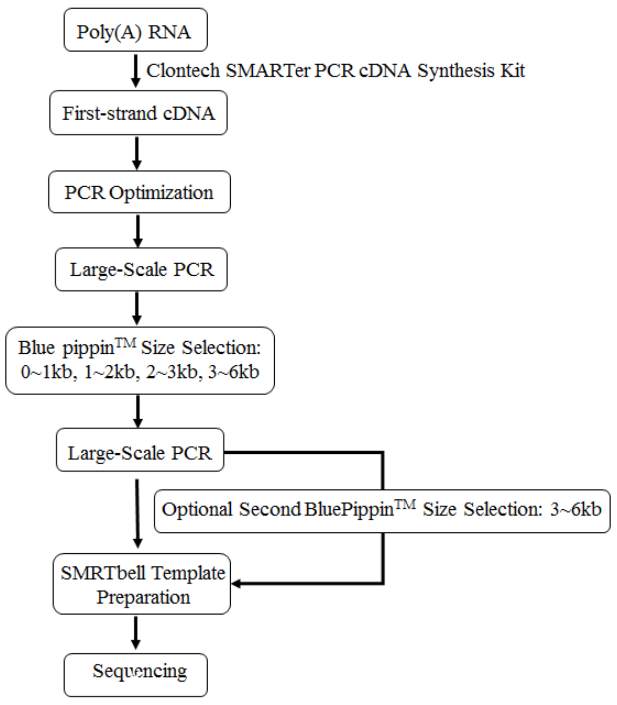

Figure S1 Experimental workflow for PacBio ISO-Seq

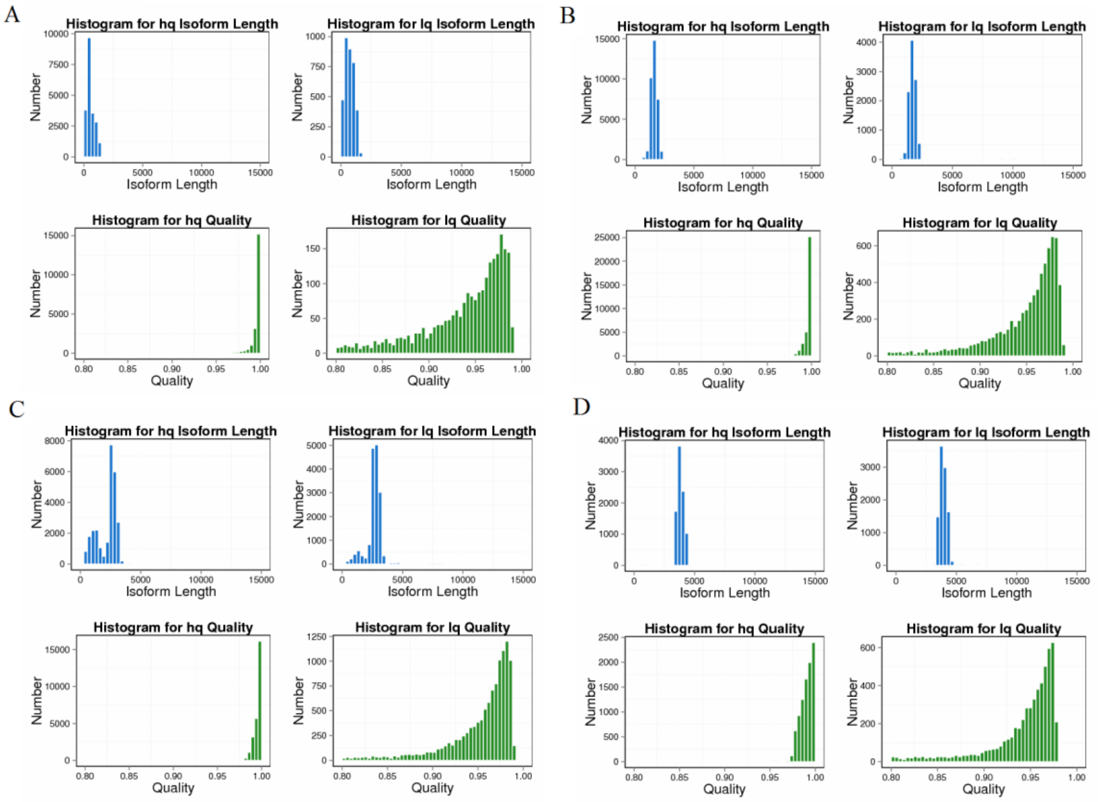

Figure S2 Length and quality distribution comparison of low QV and high QV consensus. These 4 figures illustrated the length and quality distribution of both high-quality (hq) and low-quality (lq) isoforms. We expect the lengths of the polished full-length transcript isoforms to be concordant with our size selection. a) tea.under1k; b) tea.between1k2k; c) tea.between2k3k; d) tea.between3k6k.

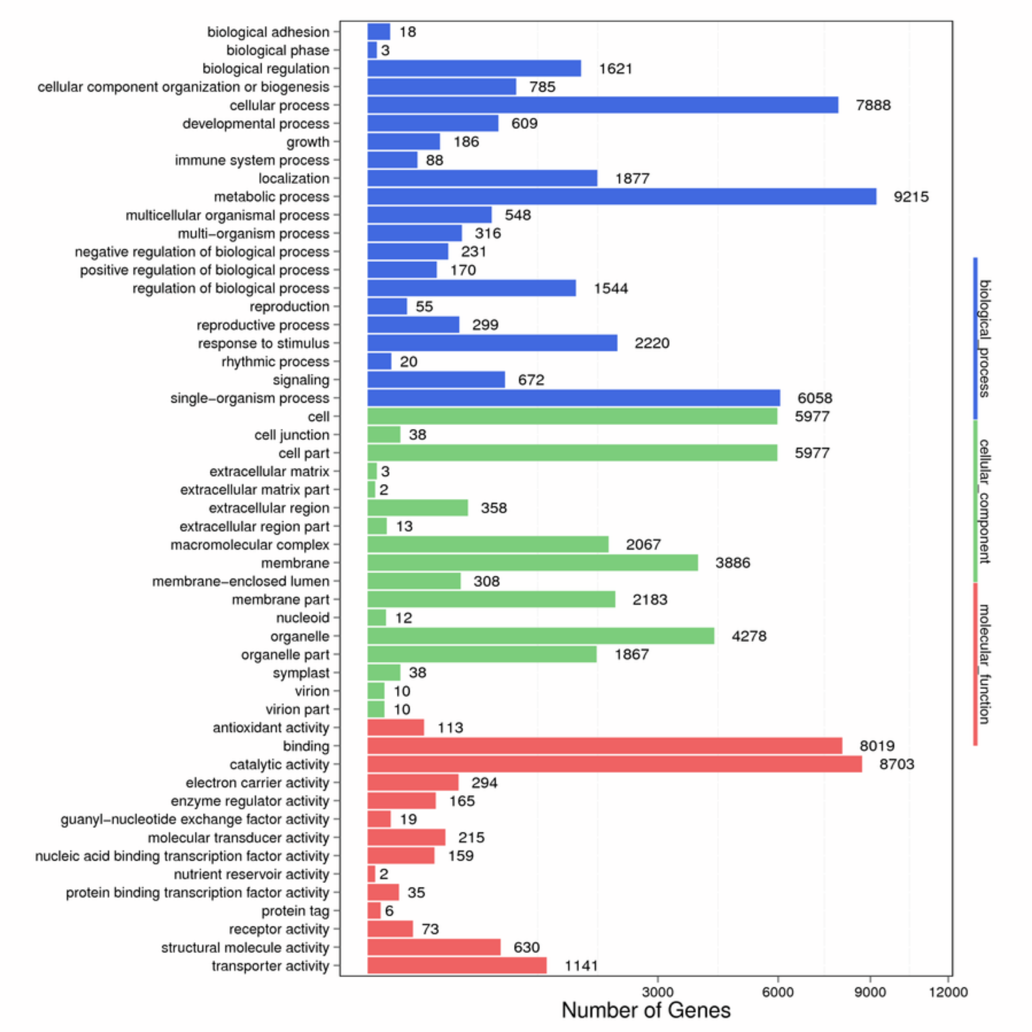

Figure S3 Functional distribution of GO annotation. X axis represents the number of trancripts. Y axis represents the Gene Ontology function category.

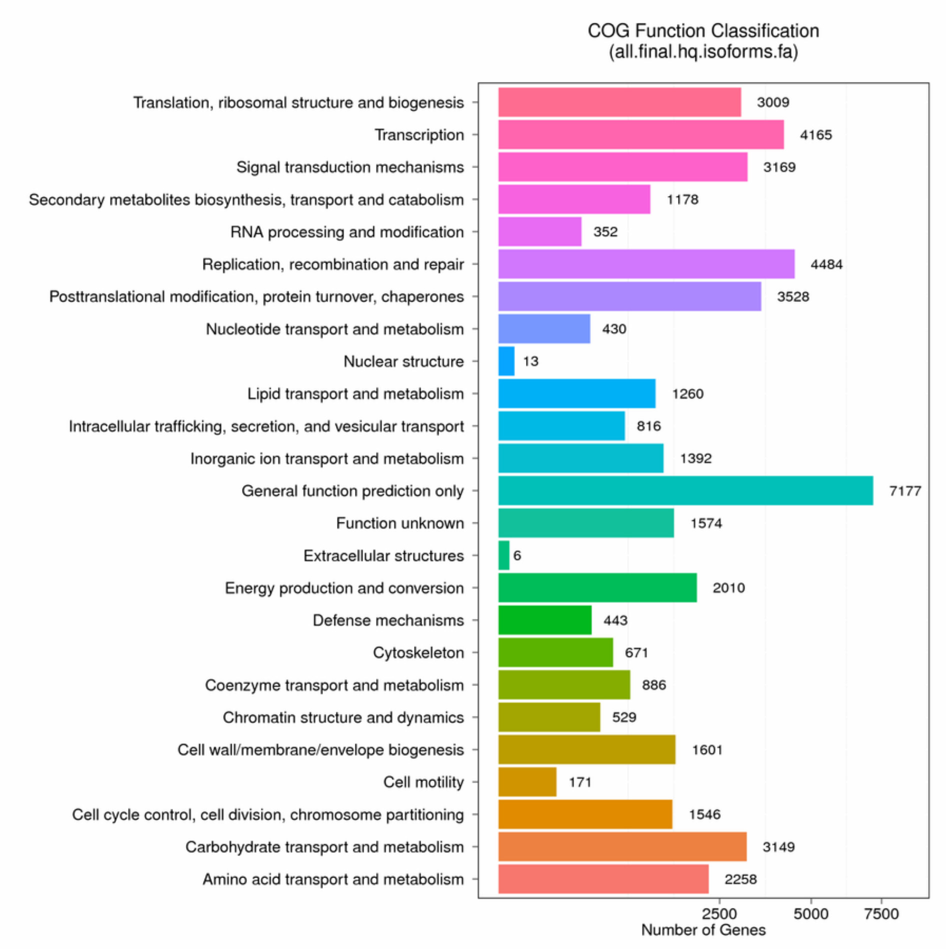

Figure S4 Functional distribution of COG annotation. X axis represents the COG function category. Y axis represents the number of transcripts.

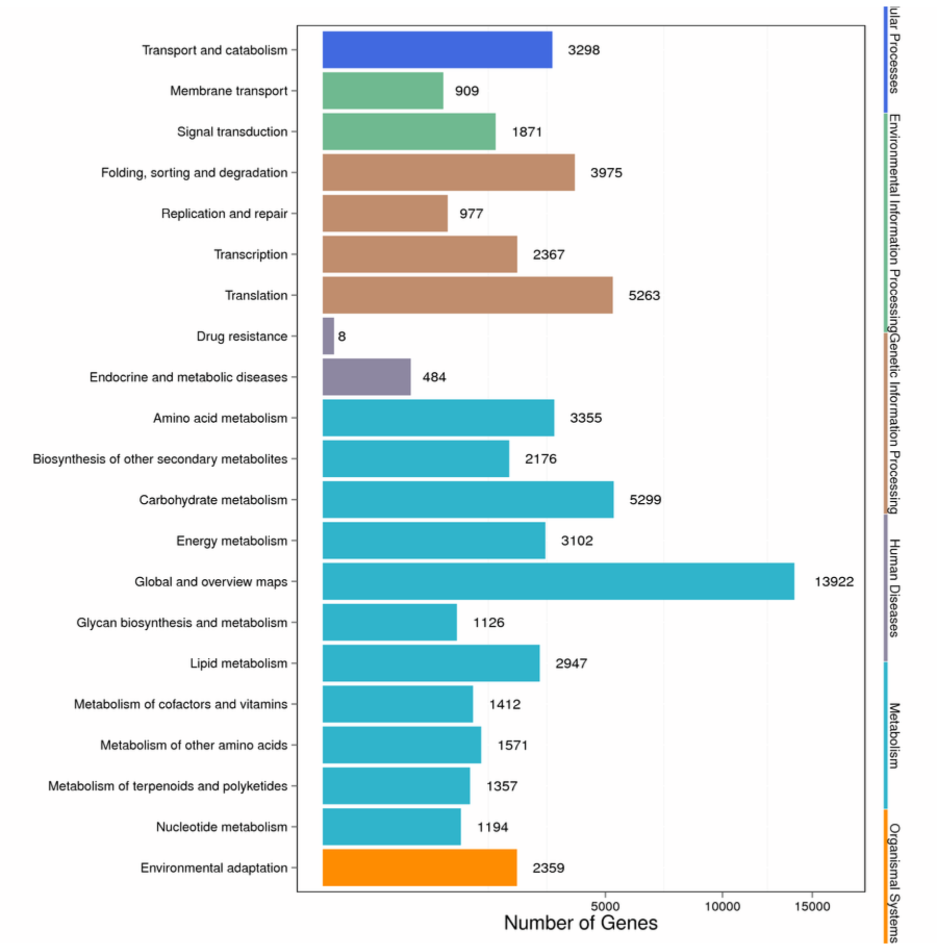

Figure S5 Functional distribution of KEGG annotation. X axis represents the number of trancripts. Y axis represents the KEGG function category.

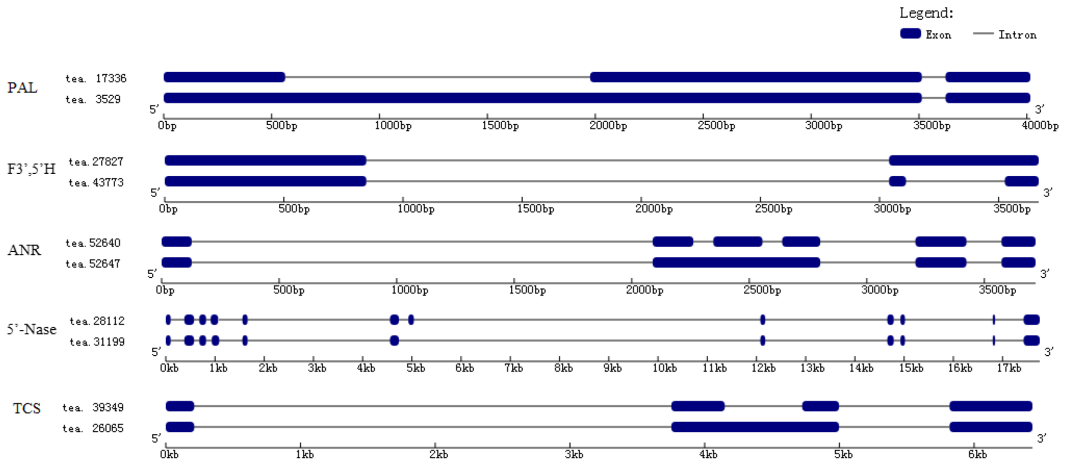

Figure S6 Alternative splicing isoforms identified in different clusters.

```
CLUSTAL multiple sequence alignment
[tea]_48033      CTGCAACATACACAAACACAATAATTAAAATTTGGTGTACTAACAATGATGACTACAGTG
CL9445.Contig2   ------------------------------------------------------------

[tea]_48033      GCTGCCCCGAGAGTCCAAAGCTTGGCGACGAGTGGGATTGAATCGATCCCAAAAGAGTAC
CL9445.Contig2   ------------------------------------------------------------

[tea]_48033      GTGAGGCCAAAAGAAGAGCTGACGGGCATCGGCAACATATTTGAAGAAGAGAAGAATGAA
CL9445.Contig2   ------------------------------------------------------------

[tea]_48033      GAAGGGCCACAAGTGCCTACAATTGACTTGAAAGACATAGACTCGGAGGTCGAGGAGGTG
CL9445.Contig2   ------------------------------------------------------------

[tea]_48033      AGAGAGAGGTGTCGGGAGGCGTTGAAGAAGGCGGCGGTGGATTGGGGTGTGATGCATTTG
CL9445.Contig2   ------------------------------------------------------------

[tea]_48033      GTGAATCATGGGATAGCGGAGGACGTGAGGGAGCGGGTGAAGGTGGCCGGAGAAGGGTTT
CL9445.Contig2   ------------------------------------------------------------

[tea]_48033      TTTGAGCAGCCGGTGGAGGAGAAGGAGAAGTATGCAAACGACCCTGATAATGGGAATCTT
CL9445.Contig2   --------------GGAGGAGAAGGAGAAGTATGCAAACGACCCTGATAATGGGAATCTT
                               **********************************************
[tea]_48033      CAAGGGTATGGGAGCAAATTGGCTAACAATGCTTGTGGTCAACTTGAATGGGAAGACTAT
CL9445.Contig2   CAAGGGTATGGGAGCAAATTGGCTAACAATGCTTGTGGTCAACTTGAATGGGAAGACTAT
                 ************************************************************
[tea]_48033      TTTTTCCACCTTGCTTACCCTGAGGACAAGTGTGACATGTCCATTTGGCCCAAGACACCA
CL9445.Contig2   TTTTTCCACCTTGCTTACCCTGAGGACAAGTGTGACATGTCCATTTGGCCCAAGACACCA
                 ************************************************************
[tea]_48033      ACCGACTACATTCCTGCAACAGTTGAGTACGCAAAGCAACTTCGAGCCCTAACTACCAAG
CL9445.Contig2   ACCGACTACATTCCTGCAACAGTTGAGTACGCAAAGCAACTTCGAGCCCTAACTACCAAG
                 ************************************************************
[tea]_48033      ACGCTCTCAATCCTATCCCTTGGCTTAGGACTAGAAGAAAACAGACTAGAGAAAGAAGTT
CL9445.Contig2   ACGCTCTCAATCCTATCCCTTGGCTTAGGACTAGAAGAAAACAGACTAGAGAAAGAAGTT
                 ************************************************************
[tea]_48033      GGAGGCAAAGAAGAGCTCCTCCTCCAAATGAAAATCAACTATTACCCAAAATGCCCTCAA
CL9445.Contig2   GGAGGCAAAGAAGAGCTCCTCCTCCAAATGAAAATCAACTATTACCCAAAATGCCCTCAA
                 ************************************************************
[tea]_48033      CCCGAGCTCGCTCTTGGGGTTGAAGCCCACACTGACTTGAGTGCAGTCTCCTTCATCCTC
CL9445.Contig2   ------------------------------------------------------------

[tea]_48033      CCCAGCATGGTCCCAGGGCTGCAACTCTTCTACGAGGGTAAGTGGATCACTGCAAAATGT
CL9445.Contig2   ------------------------------------------------------------

[tea]_48033      GTACCAAACTCTATCATCATGCTAATTGGGGACACTGTTGAAATTCTTAGTAATGGCAAG
```

```
CL9445.Contig2  ------------------------------------------------------------

[tea]_48033     TACAAGAGCATTCTTCACAGAGGACTTGTTAACAAGGAAAAGGTGAGAATTTCGTGGGCG
CL9445.Contig2  ------------------------------------------------------------

[tea]_48033     GTTTTTTGTGAGCCGCCAAAGGAGAAGATTATCTTGAAGCCATTGCCGGAGACGGTGTCA
CL9445.Contig2  ------------------------------------------------------------

[tea]_48033     GAGGCGGAGCCACCGCTGTTCCCGCCGAGGACTTTTGCTCAGCATATTCAACATAAATTG
CL9445.Contig2  ------------------------------------------------------------

[tea]_48033     TTCCGGAAATCCCAAGAACTTGGCTCTAAATAATTCTGGAAAATGCTATGCTATCGAAAT
CL9445.Contig2  ------------------------------------------------------------

[tea]_48033     TTTTATACAAAATTTGATTTCAGAATGACATGGCGGTGAGGTGAAATTTCATAAATTAAA
CL9445.Contig2  ------------------------------------------------------------

[tea]_48033     ATAAATTAATGATGGGTCTTTATGTTTTTTAATGAAAAGCCATGTTATTTTGTATGATAA
CL9445.Contig2  ------------------------------------------------------------

[tea]_48033     TTTTGTATAATTTGTCAATTTGTGTAGCATTAAGTCTAAAATTAATTGTTTGGTGGTTGG
CL9445.Contig2  ------------------------------------------------------------

[tea]_48033     TTTGTAATGTTATGGTTTGGTTTGATGTTTTAGAGTTTAATCATTGGCAGGTCTTATAGA
CL9445.Contig2  ------------------------------------------------------------

[tea]_48033     CCTTAGCCTGTATTTGTCTCAGTGCTTGTCCTTTGCCTGTCTTTTAACTACAATTTATCA
CL9445.Contig2  ------------------------------------------------------------

[tea]_48033     ATAAGATCATTTTCTT
CL9445.Contig2  ----------------

CLUSTAL multiple sequence alignment
[tea]_48033     CTGCAACATACACAAACACAATAATTAAAATTTGGTGTACTAACAATGATGACTACAGTG
CL9445.Contig3  ------------------CAATAATTAAAATTTGGTGTACTAACAATGATGACTACAGTG
                                  ******************************************
[tea]_48033     GCTGCCCCGAGAGTCCAAAGCTTGGCGACGAGTGGGATTGAATCGATCCCAAAAGAGTAC
CL9445.Contig3  GCTGCCCCGAGAGTCCAAAGCTTGGCGACGAGTGGGATTGAATCGATCCCAAAAGAGTAC
                ************************************************************
[tea]_48033     GTGAGGCCAAAAGAAGAGCTGACGGGCATCGGCAACATATTTGAAGAAGAGAAGAATGAA
CL9445.Contig3  GTGAGGCCAAAAGAAGAGCTGACGGGCATCGGCAACATATTTGAAGAAGAGAAGAATGAA
                ************************************************************
[tea]_48033     GAAGGGCCACAAGTGCCTACAATTGACTTGAAAGACATAGACTCGGAGGTCGAGGAGGTG
CL9445.Contig3  GAAGGGCCACAAGTGCCTACAATTGACTTGAAAGACATAGACTCGGAGGTCGAGGAGGTG
```

```
                ************************************************************
[tea]_48033     AGAGAGAGGTGTCGGGAGGCGTTGAAGAAGGCGGCGGTGGATTGGGGTGTGATGCATTTG
CL9445.Contig3  AGAGAGAGGTGTCGGGAGGCGTTGAAGAAGGCGGCGGTGGATTGGGGTGTGATGCATTTG
                ************************************************************
[tea]_48033     GTGAATCATGGGATAGCGGAGGACGTGAGGGAGCGGGTGAAGGTGGCCGGAGAAGGGTTT
CL9445.Contig3  GTGAATCATGGGATAGCGGAGGACGTGAGGGAGCGGGTGAAGGTGGCCGGAGAAGGGTTT
                ************************************************************
[tea]_48033     TTTGAGCAGCCGGTGGAGGAGAAGGAGAAGTATGCAAACGACCCTGATAATGGGAATCTT
CL9445.Contig3  TTTGAGCAGCCGGTGGAGGAGAAGGAGAAGTATGCA------------------------
                ************************************
[tea]_48033     CAAGGGTATGGGAGCAAATTGGCTAACAATGCTTGTGGTCAACTTGAATGGGAAGACTAT
CL9445.Contig3  ------------------------------------------------------------

[tea]_48033     TTTTTCCACCTTGCTTACCCTGAGGACAAGTGTGACATGTCCATTTGGCCCAAGACACCA
CL9445.Contig3  ------------------------------------------------------------

[tea]_48033     ACCGACTACATTCCTGCAACAGTTGAGTACGCAAAGCAACTTCGAGCCCTAACTACCAAG
CL9445.Contig3  ------------------------------------------------------------

[tea]_48033     ACGCTCTCAATCCTATCCCTTGGCTTAGGACTAGAAGAAAACAGACTAGAGAAAGAAGTT
CL9445.Contig3  ------------------------------------------------------------

[tea]_48033     GGAGGCAAAGAAGAGCTCCTCCTCCAAATGAAAATCAACTATTACCCAAAATGCCCTCAA
CL9445.Contig3  ------------------------------------------------------------

[tea]_48033     CCCGAGCTCGCTCTTGGGGTTGAAGCCCACACTGACTTGAGTGCAGTCTCCTTCATCCTC
CL9445.Contig3  ------------------------------------------------------------

[tea]_48033     CCCAGCATGGTCCCAGGGCTGCAACTCTTCTACGAGGGTAAGTGGATCACTGCAAAATGT
CL9445.Contig3  ------------------------------------------------------------

[tea]_48033     GTACCAAACTCTATCATCATGCTAATTGGGGACACTGTTGAAATTCTTAGTAATGGCAAG
CL9445.Contig3  ------------------------------------------------------------

[tea]_48033     TACAAGAGCATTCTTCACAGAGGACTTGTTAACAAGGAAAAGGTGAGAATTTCGTGGGCG
CL9445.Contig3  ------------------------------------------------------------

[tea]_48033     GTTTTTTGTGAGCCGCCAAAGGAGAAGATTATCTTGAAGCCATTGCCGGAGACGGTGTCA
CL9445.Contig3  ------------------------------------------------------------

[tea]_48033     GAGGCGGAGCCACCGCTGTTCCCGCCGAGGACTTTTGCTCAGCATATTCAACATAAATTG
CL9445.Contig3  ------------------------------------------------------------

[tea]_48033     TTCCGGAAATCCCAAGAACTTGGCTCTAAATAATTCTGGAAAATGCTATGCTATCGAAAT
```

```
CL9445.Contig3   ------------------------------------------------------------

[tea]_48033      TTTTATACAAAATTTGATTTCAGAATGACATGGCGGTGAGGTGAAATTTCATAAATTAAA
CL9445.Contig3   ------------------------------------------------------------

[tea]_48033      ATAAATTAATGATGGGTCTTTATGTTTTTTAATGAAAAGCCATGTTATTTTGTATGATAA
CL9445.Contig3   ------------------------------------------------------------

[tea]_48033      TTTTGTATAATTTGTCAATTTGTGTAGCATTAAGTCTAAAATTAATTGTTTGGTGGTTGG
CL9445.Contig3   ------------------------------------------------------------

[tea]_48033      TTTGTAATGTTATGGTTTGGTTTGATGTTTTAGAGTTTAATCATTGGCAGGTCTTATAGA
CL9445.Contig3   ------------------------------------------------------------

[tea]_48033      CCTTAGCCTGTATTTGTCTCAGTGCTTGTCCTTTGCCTGTCTTTTAACTACAATTTATCA
CL9445.Contig3   ------------------------------------------------------------

[tea]_48033      ATAAGATCATTTTCTT
CL9445.Contig3   ----------------

CLUSTAL multiple sequence alignment
[tea]_48033      CTGCAACATACACAAACACAATAATTAAAATTTGGTGTACTAACAATGATGACTACAGTG
Unigene6902      ------------------------------------------------------------

[tea]_48033      GCTGCCCCGAGAGTCCAAAGCTTGGCGACGAGTGGGATTGAATCGATCCCAAAAGAGTAC
Unigene6902      ------------------------------------------------------------

[tea]_48033      GTGAGGCCAAAAGAAGAGCTGACGGGCATCGGCAACATATTTGAAGAAGAGAAGAATGAA
Unigene6902      ------------------------------------------------------------

[tea]_48033      GAAGGGCCACAAGTGCCTACAATTGACTTGAAAGACATAGACTCGGAGGTCGAGGAGGTG
Unigene6902      ------------------------------------------------------------

[tea]_48033      AGAGAGAGGTGTCGGGAGGCGTTGAAGAAGGCGGCGGTGGATTGGGGTGTGATGCATTTG
Unigene6902      ------------------------------------------------------------

[tea]_48033      GTGAATCATGGGATAGCGGAGGACGTGAGGGAGCGGGTGAAGGTGGCCGGAGAAGGGTTT
Unigene6902      ------------------------------------------------------------

[tea]_48033      TTTGAGCAGCCGGTGGAGGAGAAGGAGAAGTATGCAAACGACCCTGATAATGGGAATCTT
Unigene6902      ------------------------------------------------------------

[tea]_48033      CAAGGGTATGGGAGCAAATTGGCTAACAATGCTTGTGGTCAACTTGAATGGGAAGACTAT
Unigene6902      ------------------------------------------------------------
```

```
[tea]_48033     TTTTTCCACCTTGCTTACCCTGAGGACAAGTGTGACATGTCCATTTGGCCCAAGACACCA
Unigene6902     ------------------------------------------------------------

[tea]_48033     ACCGACTACATTCCTGCAACAGTTGAGTACGCAAAGCAACTTCGAGCCCTAACTACCAAG
Unigene6902     ------------------------------------------------------------

[tea]_48033     ACGCTCTCAATCCTATCCCTTGGCTTAGGACTAGAAGAAAACAGACTAGAGAAAGAAGTT
Unigene6902     ------------------------------------------------------------

[tea]_48033     GGAGGCAAAGAAGAGCTCCTCCTCCAAATGAAAATCAACTATTACCCAAAATGCCCTCAA
Unigene6902     --------------------------------------CTATTACCCAAAATGCCCTCAA
                                                      **********************
[tea]_48033     CCCGAGCTCGCTCTTGGGGTTGAAGCCCACACTGACTTGAGTGCAGTCTCCTTCATCCTC
Unigene6902     CCCGAGCTCGCTCTTGGGGTTGAAGCCCACACTGACTTGAGTGCAGTCTCCTTCATCCTC
                ************************************************************
[tea]_48033     CCCAGCATGGTCCCAGGGCTGCAACTCTTCTACGAGGGTAAGTGGATCACTGCAAAATGT
Unigene6902     CCCAGCATGGTCCCAGGGCTGCAACTCTTCTACGAGGGTAAGTGGATCACTGCAAAATGT
                ************************************************************
[tea]_48033     GTACCAAACTCTATCATCATGCTAATTGGGGACACTGTTGAAATTCTTAGTAATGGCAAG
Unigene6902     GTACCAAACTCTATCATCATGCTAATTGGGGACACTGTTGAAATTCTTAGTAATGGCAAG
                ************************************************************
[tea]_48033     TACAAGAGCATTCTTCACAGAGGACTTGTTAACAAGGAAAAGGTGAGAATTTCGTGGGCG
Unigene6902     TACAAGAGCATTCTTCACAGAGGACTTGTTAACAAGGAAAAGGTGAGAATTTCGT-----
                *******************************************************
[tea]_48033     GTTTTTGTGAGCCGCCAAAGGAGAAGATTATCTTGAAGCCATTGCCGGAGACGGTGTCA
Unigene6902     ------------------------------------------------------------

[tea]_48033     GAGGCGGAGCCACCGCTGTTCCCGCCGAGGACTTTTGCTCAGCATATTCAACATAAATTG
Unigene6902     ------------------------------------------------------------

[tea]_48033     TTCCGGAAATCCCAAGAACTTGGCTCTAAATAATTCTGGAAAATGCTATGCTATCGAAAT
Unigene6902     ------------------------------------------------------------

[tea]_48033     TTTTATACAAAATTTGATTTCAGAATGACATGGCGGTGAGGTGAAATTTCATAAATTAAA
Unigene6902     ------------------------------------------------------------

[tea]_48033     ATAAATTAATGATGGGTCTTTATGTTTTTTAATGAAAAGCCATGTTATTTTGTATGATAA
Unigene6902     ------------------------------------------------------------

[tea]_48033     TTTTGTATAATTTGTCAATTTGTGTAGCATTAAGTCTAAAATTAATTGTTTGGTGGTTGG
Unigene6902     ------------------------------------------------------------

[tea]_48033     TTTGTAATGTTATGGTTTGGTTTGATGTTTTAGAGTTTAATCATTGGCAGGTCTTATAGA
```

```
Unigene6902       ------------------------------------------------------------

[tea]_48033       CCTTAGCCTGTATTTGTCTCAGTGCTTGTCCTTTGCCTGTCTTTTAACTACAATTTATCA
Unigene6902       ------------------------------------------------------------

[tea]_48033       ATAAGATCATTTTCTT
Unigene6902       ----------------

CLUSTAL multiple sequence alignment
[tea]_48033       CTGCAACATACACAAACACAATAATTAAAATTTGGTGTACTAACAATGATGACTACAGTG
Unigene22214      ------------------------------------------------------------

[tea]_48033       GCTGCCCCGAGAGTCCAAAGCTTGGCGACGAGTGGGATTGAATCGATCCCAAAAGAGTAC
Unigene22214      ------------------------------------------------------------

[tea]_48033       GTGAGGCCAAAAGAAGAGCTGACGGGCATCGGCAACATATTTGAAGAAGAGAAGAATGAA
Unigene22214      ------------------------------------------------------------

[tea]_48033       GAAGGGCCACAAGTGCCTACAATTGACTTGAAAGACATAGACTCGGAGGTCGAGGAGGTG
Unigene22214      ------------------------------------------------------------

[tea]_48033       AGAGAGAGGTGTCGGGAGGCGTTGAAGAAGGCGGCGGTGGATTGGGGTGTGATGCATTTG
Unigene22214      ------------------------------------------------------------

[tea]_48033       GTGAATCATGGGATAGCGGAGGACGTGAGGGAGCGGGTGAAGGTGGCCGGAGAAGGGTTT
Unigene22214      ------------------------------------------------------------

[tea]_48033       TTTGAGCAGCCGGTGGAGGAGAAGGAGAAGTATGCAAACGACCCTGATAATGGGAATCTT
Unigene22214      ------------------------------------------------------------

[tea]_48033       CAAGGGTATGGGAGCAAATTGGCTAACAATGCTTGTGGTCAACTTGAATGGGAAGACTAT
Unigene22214      ------------------------------------------------------------

[tea]_48033       TTTTTCCACCTTGCTTACCCTGAGGACAAGTGTGACATGTCCATTTGGCCCAAGACACCA
Unigene22214      ------------------------------------------------------------

[tea]_48033       ACCGACTACATTCCTGCAACAGTTGAGTACGCAAAGCAACTTCGAGCCCTAACTACCAAG
Unigene22214      ------------------------------------------------------------

[tea]_48033       ACGCTCTCAATCCTATCCCTTGGCTTAGGACTAGAAGAAAACAGACTAGAGAAAGAAGTT
Unigene22214      ------------------------------------------------------------

[tea]_48033       GGAGGCAAAGAAGAGCTCCTCCTCCAAATGAAAATCAACTATTACCCAAAATGCCCTCAA
Unigene22214      ------------------------------------------------------------
```

```
[tea]_48033     CCCGAGCTCGCTCTTGGGGTTGAAGCCCACACTGACTTGAGTGCAGTCTCCTTCATCCTC
Unigene22214    ------------------------------------------------------------

[tea]_48033     CCCAGCATGGTCCCAGGGCTGCAACTCTTCTACGAGGGTAAGTGGATCACTGCAAAATGT
Unigene22214    ------------------------------------------------------------

[tea]_48033     GTACCAAACTCTATCATCATGCTAATTGGGGACACTGTTGAAATTCTTAGTAATGGCAAG
Unigene22214    ------------------------------------------------------------

[tea]_48033     TACAAGAGCATTCTTCACAGAGGACTTGTTAACAAGGAAAAGGTGAGAATTTCGTGGGCG
Unigene22214    ------------------------CTTGTTAACAAGGAAAAGGTGAGAATTTCGTGGGCG
                                        ************************************
[tea]_48033     GTTTTTTGTGAGCCGCCAAAGGAGAAGATTATCTTGAAGCCATTGCCGGAGACGGTGTCA
Unigene22214    GTTTTTTGTGAGCCGCCAAAGGAGAAGATTATCTTGAAGCCATTGCCGGAGACGGTGTCA
                ************************************************************
[tea]_48033     GAGGCGGAGCCACCGCTGTTCCCGCCGAGGACTTTTGCTCAGCATATTCAACATAAATTG
Unigene22214    GAGGCGGAGCCACCGCTGTTCCCGCCGAGGACTTTTGCTCAGCATATTCAACATAAATTG
                ************************************************************
[tea]_48033     TTCCGGAAATCCCAAGAACTTGGCTCTAAATAATTCTGGAAAATGCTATGCTATCGAAAT
Unigene22214    TTCCGGAAATCCCAAGAACTTGGCTCTAAATAATTCTGGAAAATGCTATGCTATCGAAAT
                ************************************************************
[tea]_48033     TTTTATACAAAATTTGATTTCAGAATGACATGGCGGTGAGGTGAAATTTCATAAATTAAA
Unigene22214    TTTTATACAAAATTTGATTTCAGAATGACATGGCGGTGAGGTGAAATTTCATAAATTAAA
                ************************************************************
[tea]_48033     ATAAATTAATGATGGGTCTTTATGTTTTTTAATGAAAAGCCATGTTATTTTGTATGATAA
Unigene22214    ATAAATTAATGATGGG--------------------------------------------
                ****************
[tea]_48033     TTTTGTATAATTTGTCAATTTGTGTAGCATTAAGTCTAAAATTAATTGTTTGGTGGTTGG
Unigene22214    ------------------------------------------------------------

[tea]_48033     TTTGTAATGTTATGGTTTGGTTTGATGTTTTAGAGTTTAATCATTGGCAGGTCTTATAGA
Unigene22214    ------------------------------------------------------------

[tea]_48033     CCTTAGCCTGTATTTGTCTCAGTGCTTGTCCTTTGCCTGTCTTTTAACTACAATTTATCA
Unigene22214    ------------------------------------------------------------

[tea]_48033     ATAAGATCATTTTCTT
Unigene22214    ----------------
```

Figure S7 The alignment of several short-seq transcripts with same long-read gene.
